# Dataset for estimating occurrence probability of causations for plugged, abandoned and decommissioned oil and gas wells

**DOI:** 10.1016/j.dib.2020.105988

**Published:** 2020-07-05

**Authors:** Ahmed Babaleye, Rafet Emek Kurt, Faisal Khan

**Affiliations:** a Department of Naval Architecture, Ocean and Marine Engineering, University of Strathclyde, Glasgow, United Kingdom; b Centre for Risk, Integrity and Safety Engineering (C-RISE), Faculty of Engineering and Applied Science, Memorial University of Newfoundland, St. John's, NL A1B 3X5, Canada

**Keywords:** Hierarchical Bayesian model, Failure analysis, Well plugging and abandonment, Decommissioning

## Abstract

This article contains the dataset on the failure frequencies of the barrier and mechanical plugs in place within the hydrocarbon-containing wellbore during plugging and abandonment operation. The interpretation and application of this data can be found in the research article (“https://doi.org/10.1016/j.psep.2019.09.015” Babaleye et al., 2019). These datasets were collected through a comprehensive hazard identification technique workshop involving 10 engineers and academics with considerable years of field experience. The data were collected based on how likely it is for each causation to occur and these likelihoods are ranked from 1 to 10. The process is experience-driven and is complemented by a 1–10 rating of the duration of leak of hydrocarbon before remediation, should the leak reach the mudline. The ranked data was a representative of raw failure data (failure rate or mean time to failure (MTTF)) for each causation and are coded in MATLAB using gamma distribution based on hierarchical Bayesian analysis. The dataset offers unique opportunity for reuse due to its accessibility and discreteness.

## Specifications Table

**Table:** 

**Subject**	Safety, Risk, Reliability and Quality
**Specific subject area**	Ocean Engineering
**Type of data**	Table Figure
**How data was acquired**	Workshop, Hazard identification, Risk factors ranking techniques
**Data format**	Raw Analyzed (MATLAB)
**Parameters for data collection**	The limited knowledge of the well failure modes, unknown reservoir condition, the nature and type of well (i.e. whether it is oil and/or gas), severity of the wellbore (i.e. sour or nonsour well) and structural adequacy of the well casings are all considered variables of uncertainty.
**Description of data collection**	The data provides alternative source for obtaining the failure data to conduct a probabilistic risk analysis of abandoned and decommissioned wells. The datasets were obtained through plugging and abandonment workshop involving 10 engineers and academics with considerable knowledge of abandoned well safety issues and field experience.
**Data source location**	Institution: University of Strathclyde City: Glasgow Country: United Kingdom.
**Data accessibility**	Repository: Mendeley, StrathCloud URL: http://dx.doi.org/10.17632/mz3khsphdb.1

## Value of the data

• The dataset presented here can provide relevant information for quantifying the risks of plugged, abandoned and decommissioned oil and gas wells. The data describe the ranking from 10 engineers and academics with considerable field experience. It represents equivalent failure data collected from different sources.
• The data obtained for the plugged and abandoned failure analysis can be used as a basis for estimating the occurrence probability of each causation leading to well decommissioning failure.
• The data can be used to conduct probabilistic risk analysis of complex well plugging and abandonment operational failure, or the lack of it thereof.
• Both offshore industry professionals and research talents can adopt this dataset to inform their safety culture and failure analysis modelling.
• The data can be used as a basis in the design of experiments (DoE) to reduce the variability associated with data paucity.

## 1 Data description

This data represents the number of failures recorded for each mechanical or barrier plug implemented within the wellbore of a plugged and abandoned well. The described dataset is applied to the 22/30c-G4 Well Platform case study. The well is located in the middle of the North Sea between Scotland and Norway, approximately 240 km East of Aberdeen. Cessation of production is already in place and the well was undergoing plugging and abandonment operation when natural gas in enormous quantity began to leak into the wellbore due to an abrupt pressure differential that could not be bled off in good time. The principal particulars of the well are as shown in Table 1 below.

**Table 1:** Case study principal particulars.

Platform Particulars	
Reservoir condition	HT/HP
Ocean depth	90 m
Reservoir depth	5500 m
Reservoir temperature	190 °C
Reservoir pressure	1100 bar
Fluid type	Natural gas
Fluid severity	Sour

In the event of data acquisition, basic events capable of nonlinear interactions and initiating the overall failure of the permanent abandonment activity was defined and assigned identifiers as contained in Table 2, to guide risk assessors in the allocation of applicable weightage.

**Table 2:** Identified hazards for permanent abandonment failure.

Events Identifier	Event Description
B_1.1_	Pressure differentials
B_1.2_	Injection into nearby walls
B_2_	Leak through lower/primary plug
B_3.1_	Prolong exposure to migrating fluid
B_3.2.1_	Formation fluids load effect
B_3.2.2_	Geological forces
B_4_	Leak through lower/primary plug
B_5.1_	De-bonding of plug & casing
B_5.2,_ B_6.2,_ B_7.2_	Annulus barrier degradation
B_6.1.1_	Inadequate barrier density
B_6.1.2_	Loss of barrier
B_7.1.1_	Poor mud removal
B_7.1.2_	Barrier shrinkage

For every failure frequency reported, a corresponding number of trials for the leakage duration was also noted. The data reflects the subjectivity of expert judgements that is often relied on when process knowledge is limited, or complete hazards cannot be captured. Through hazard identification workshop, each causation contributing to the overall failure of abandoned well, characterized by the leak of hydrocarbon through the mudline, is ranked based on their likelihood of occurrence as deemed by each respondent [1]. The collected failure data and their corresponding trials (representing the duration of the hydrocarbon leak to mudline) including those obtained for the safety barriers in place are presented in Table 2 [1] and Table 3 below, respectively, in accordance with the procedure demonstrated in [2].

**Table 3:** Safety barriers failure precursor data.

Source	Demands (*N_i_*)	HDS	IPS	FAS	AaS	EES
1	11	–	1	1	–	–
2	13	–	2	–	2	–
3	13	–	1	1	–	–
4	18	–	–	–	1	–
5	11	3	–	–	–	–
6	12	8	–	1	–	–
7	15	4	4	4	–	–
8	12	7	3	–	–	–
9	16	2	–	–	–	–
10	20	1	–	–	–	1

The data indicates the expected number of times the barrier plugs would fail and establishes the relationship between successive failures of interacting causations. The data are then coded using hierarchical Bayesian analysis to estimate the predictive posterior distributions (PPDs) for each causation. Each PPD represents the mean probability of failure that can be used to quantify the overall risk of well plugging and abandonment failure. As the ranking varies significantly between respondents, which is typical of expert opinions from source-to-source, the probability distribution curves have different densities as seen in Figure 7 shown in [1].

## 2 Experimental design, materials and methods

**Fig. 1:**
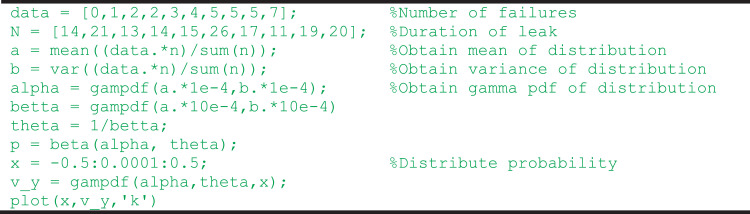
MATLAB code for probability estimation.

The data was obtained through the workshop on a presented real-life decommissioning and abandonment operation from the Elgin platform well failure [1]. The dataset depicts the number of failures of a barrier plug and the plugging and abandonment trials conducted. The collection criteria included the overall knowledge of the accident evolution leading to failure of the abandonment operation, the opinion of each participant (expert judgement) ranked on a scale of 1–10, and the number of demands (duration of the leak) for which the operation is continued before the well is killed, were on a scale of 1–5. To obtain the failure probability of each causation factor, the data were aggregated based on hierarchical Bayesian approach (HBA) and coded in MATLAB (version R2018a) as shown in Fig. 1. In Fig. 1, the number of failures and their corresponding demands (described as duration of leak) are aggregated to yield the distribution average and extent of variation amongst data – designated as a and b, respectively. The shape and scale parameters represented as ‘alpha’ and ‘betta’ are assumed to be gamma distribution and the failure probability is obtained as a beta function due to its conjugate pair. For the sake of clarity, the probability density function is spread over the interval [−0.5 ≤ *x* ≤ 0.5] at 1e-4 increment.

## Declaration of Competing Interest

The authors declare that they have no known competing financial interests or personal relationships which have, or could be perceived to have, influenced the data collection and processing reported in this article.

## Appendix Supplementary materials

Image, application 1
